# Another face of violence against women: Infertility

**DOI:** 10.12669/pjms.334.12862

**Published:** 2017

**Authors:** Rusen Ozturk, Aylin Taner, Sezer Er Guneri, Bulent Yilmaz

**Affiliations:** 1 Rusen Ozturk, Ph.D. Research Assistant, Department of Gynecologic and Obstetric Nursing, Ege University Faculty of Nursing, Izmir, Turkey; 2 Aylin Taner, RN. MSc Student, Department of Gynecologic and Obstetric Nursing, Ege University Faculty of Nursing, Izmir, Turkey; 3 Sezer Er Guneri, Ph.D. Assistant Professor, Department of Gynecologic and Obstetric Nursing, Ege University Faculty of Nursing, Izmir, Turkey; 4 Bulent Yilmaz, Associate Professor, Medical Doctor, Tepecik Education and Research Hospital, Women’s Diseases and Birth, Turkey

**Keywords:** Infertile women, Domestic violence, Nursing

## Abstract

**Background & Objective:**

Violence against women is a widespread problem and has serious implications on women’s health. Infertility, in many ways, is a very stressful condition that affect social and marital life of a couple; moreover, compared to fertile women, infertile women are twice as vulnerable against violence. Our objective was to determine the prevalence of violence and define the effect of infertility on violence on women receiving infertility treatment.

**Methods:**

Descriptive and cross-sectional study was carried out 301 infertile women between November 2015 and August 2016 in a state hospital, Izmir. Data were collected as “Sociodemographic Characteristics Form” and “Infertile Women’s Exposure to Violence Determination Scale”.

**Results:**

The mean age of women was 31.77±5.46 years; the average duration of marriage was 6.93±4.53 years. About 32.5% of women stated that they have suffered from violence throughout their lives and 4.7% of women were still suffering from violence, while 5.0% of women were subjected to violence after infertility was diagnosed.

**Conclusion:**

It is an encouraging finding that infertile women have a low exposure to violence. However, despite a low violence rate, there is an increase in violence toward women who have been diagnosed with infertility.

## INTRODUCTION

Having a child is a very important goal for most couples. Therefore, a diagnosis of infertility often causes a state of crisis because it negatively affects a couple’s relationship.^1^ The worldwide infertility rate is 8–12%, while this rate is 10–20% in Turkey.^2^ Infertility shows itself as a sudden and unexpected life crisis, and a prolonged diagnostic and treatment process, and the limitations in the adaptation process lead to serious stress.^1^,^3^

Violence affects the lives of millions of infertile women worldwide, regardless of their socioeconomic and educational level.^4^ Bibi et al. (2014) found that 20% of women who suffered from violence were subjected to violence due to infertility; Kaur (2014) found that 7% of women considered infertility a factor contributing to violence.^5^,^6^ Previous studies have reported that in the presence of infertility, the prevalence of violence toward women from their husbands or partners ranged between 1.8% and 77.8% in the world.^3^,^4^,^7^ Psychological violence was the most frequently seen type of violence in infertile women.^3^,^7^ Because of the cultural perception that infertility is the problem of women alone, violence against women is more common in the male-dominated social structure. The only study in our country in this regard reflects the eastern region with a traditional structure.^8^ In this respect, it is important to examine the prevalence of violence in infertile women in the western region. It is also essential to routinely screen infertile women, determine the likelihood of their exposure to domestic violence, and, if necessary, provide early intervention to reduce violence and possible harm. For this purpose, research was planned using a descriptive design to determine the conditions of exposure to violence and affected factors among women receiving infertility treatment in the western region of Turkey.

## METHODS

Between November 1, 2015 and August 1, 2016, the study was conducted on 301 infertile women at the infertility department of the Tepecik Training and Research Hospital, which is the only in vitro fertilization center in the Aegean region affiliated with the Ministry of Health. The number of infertile women who were treated in the hospital in 2014 was 865. By calculating 95% confidence interval using a population-based formula, it was determined that 267 women should be included in the sample. The sample selection criteria were as follows: (1) women who were diagnosed with primary infertility, (2) attended the selected hospital for treatment, (3) were 18 years and older, (4) could speak the Turkish language, and (5) agreed to participate in the study. A written consent was obtained from all the women after explaining the purpose and method of the study, and guarantee was given for privacy of answers. After a questionnaire on sociodemographic characteristics was filled by the researcher using a face-to-face interview, it was expected that the questions on violence would be answered by the women themselves. The Ethics Committee of the Ege University of Nursing Faculty approved the study protocol.

### Variables

For the collection of research data, we used a sociodemographic characteristics form, which consisted of 28 questions, and the Infertile Women’s Exposure to Violence Determination Scale (IWEVDS). This scale was developed by Onat (2014) to determine violence against infertile women and consisted of 31 questions with 5 likert-type possible answers (min:31–max:155 scores). Higher scores indicate a higher, more frequent exposure to violence. The scale had five domains: “domestic violence,” “social pressure,” “punishment,” “exposure to traditional practices,” and “exclusion”. Cronbach’s alpha coefficient of the scale was 0.96.^9^ In the present study, the Cronbach’s alpha coefficient was 0.91 and was considerably higher.

### Analysis

For data analysis, the SPSS 15.0 package program was used, and sociodemographic characteristics were calculated as frequency and percentage. To determine the data’s suitability to normal distribution, the Shapiro-Wilk test/Kolmogrov-Smirnov (K-S) (p<0.05) test was performed. Since the data did not show suitability to normal distribution, the Mann-Whitney U test and the Kruskal-Wallis test were used.

## RESULTS

### Sociodemographic Variables

Women’s mean age was 31.77±5.46 years (min: 19, max:46); 60% of them were married with mutual agreement; the average duration of marriage was 6.93±4.53 years.

The average time of diagnosis was 3.85±4.14 years; the mean duration of treatment was 3.21±3.38 years. The causes of infertility originated from the woman (34.2%), were idiopathic (37.2%), or originated from the man (16.3%). Of the total participants, 60.1% of the women had received treatment before; the mean number of insemination was 1.08±1.38, the mean number of IVF was 0.69±1.10, and 71.1% of the women were receiving IVF treatment at the time of the study.

Of the women in the study, 32.5% of them stated that they have suffered from violence throughout their lives; 62.2% of this group had suffered from domestic violence once or twice, while 15.3% of them had suffered six or more times. In 35.1% of the cases violence came from environment/friends/relatives; 38.7% of violence was verbal, 31.9% physical, and 21.8% emotional. Some women stated that they were still suffering from violence (4.7%), that they were exposed to violence after infertility was diagnosed (6.6%), and that the infertility diagnosis had increased the violence (5.0%).

### Findings Related to the Infertile Women’s Exposure to Violence Determination Scale

The mean score received from the IWEVDS was 38.74±11.49 (min:31, max:106) (Table-I). A significant difference was found between the total scale scores and age groups, educational level, economic status, mother’s educational level, previously received treatment, and current treatment type (p<0.05). The possibility of exposure to violence was found to be higher in women within the 24–28 age group, women who have low economic status, women who were literate, women who had previously received treatment and women who underwent IVF treatment. A significant difference was not observed between the total violence score and the women’s employment status, spouses’ educational levels, types of marriage, the places that they lived the longest, places of birth, the individuals with whom they live, and infertility causes (p>0.05) (Table-II). However, a negative relationship was found between the mean scale score and the age of marriage (r= -0.190, p = 0.001), while a positive relationship was found with the duration of marriage (r= -0.134, p=0,020). There was also a significant negative relationship between the mean scale score and monthly income (r= -0.121, p=0,036).

**Table-I:** Distribution of Infertile Women’s Exposure to Violence Determination Scale (IWEVDS)’s Subscale and Total Scale Scores.

	*n*	*Min*	*Max*	x̅	*Sd*
Infertile Women’s Exposure to Violence Determination Scale Total Score	301	31.00	106.00	38.7409	11.48503
Domestic violence domain		11.00	34.00	12.4884	3.22036
Social pressure domain		7.00	25.00	8.1362	2.30898
Punishment domain		6.00	25.00	8.0233	3.22947
Exposure to traditional practices domain		4.00	19.00	6.4884	2.93553
Exclusion domain		3.00	13.00	3.6047	1.43056

**Table-II:** Effective factors of infertile women’s exposure to violence determination scale.

	*n*	*%*	*x̅*	*Sd*	*Mean Rank*	*p*
***Age Group***						
19–23	23	7.6	37.26	8.2	145.8	X^2^= 9,713
24–28	68	22.6	41.99	14.72	171.92	P = 0.021
29–33	79	26.2	39.34	10.55	161.1	
34 and up	131	43.5	36.95	10.26	134.96	
***Education level of women***					
Literate	25	8.3	43.2	15.63	193.12	X^2^= 8.108
Primary education	114	37.9	38.99	11.14	154.65	P = 0.044
High school	110	36.5	37.75	10.99	140.21	
University and higher education	52	17.3	38.15	10.75	145.57	
***Employment status of women***					
Employed	200	66.4	39.4	12	156.37	U = -9025.500
Unemployed	101	33.6	37.44	10.3	140.16	P = 0.128
***Economic Status***					
Good	56	18.6	36.53	10.49	125.35	X^2^= 6.247
Moderate	207	68.8	39,04	11.29	155.94	P = 0.044
Weak	38	12.6	40.37	13.65	161.87	
***Type of Marriage***					
Mutual agreement	181	60.1	38.1	11.11	143.81	X^2^= 5.425
Arranged voluntarily marriage	104	34.6	39.08	10.99	157.81	P = 0.143
Forced marriage	2	0.7	38	9.9	145.75	
Eloped	14	4.7	44.64	18.04	194.14	
***Area where they lived most of their lives***					
Bay	52	17.3	41.58	12.02	179.87	X^2^= 7.179
District	52	17.3	37.79	11.79	141.19	P = 0.066
City	69	22.9	41.29	16.68	146.21	
Metropolis	128	42.5	36.6	6.07	145.84	
***Place of Birth***					
Bay	51	16.9	41.76	1.67	174.03	X^2^= 6.575
District	85	28.2	38.21	1.63	143.28	P = 0.087
City	70	23.3	40.36	2	159	
Metropolis	95	31.6	36.4	2.53	139.65	
***Type of Family***					
Nuclear	254	84.4	38.1575	9.86	149.46	X^2^= 1.812
Extended (husband and his family)	41	13.6	41.5122	15.7	164.78	P = 0.404
Extended (husband and her family)	6	2	44.5	30.15	121.92	
***Caused by Infertility Factors***					
Female Factor	103	34.2	39.38	12.76	150.36	X^2^= 3.931
Male Factor	49	16.3	40.51	13.08	167.62	P = 0.239
Both Partners	37	12.3	36.89	5.2	161.49	
Idiopathic	112	37.2	37.99	10.98	140.85	
***Previously Receiving Treatment***					
Yes	181	60.1	39.2	10.5	160.67	U = 9109.500
No	120	39.9	38.05	12.84	136.41	P = 0.017
***Current Treatment***					
Insemination	46	15.3	37.67	12.06	134.04	X^2^= 8.613
IVF	214	71.1	39.67	12.09	160.09	P = 0.013
Medication	41	13.6	35.1	5.28	122.6	

## DISCUSSION

In the present study, it was established that one-third of the infertile women were victims of domestic violence, and infertility diagnosis increased the incidence of violence. It has been reported that women who are exposed to domestic violence as a result of infertility are two times more defenseless than those having children.^3^,^4^,^7^,^8^,^10^ Previous studies have shown that 1.8% of women in Hong Kong, 41.6% in Nigeria, 64% in Pakistan, 61.8% in Iran, and 77.8% women in India experienced domestic violence in their marriages.^3^,^4^,^7^,^11^,^12^ In a study carried out in Turkey (2009), 33.6% of infertile women reported domestic violence because of infertility.^11^ Even though that this study revealed a lower frequency of violence compared to other studies, it is still notable that the infertility diagnosis increased the incidence of domestic violence.

Verbal, emotional, and physical violence are the types of violence that infertile women were exposed to most often in our study. Ardabily et al.(2011) described the violence as psychological (33.8%) and physical (14%); Leung et al.(2003) described it as being exposed to emotional violence (55.6%); Ameh et al. (2007) found a psychological violence rate of 51.5%; Sami and Ali (2011) described it as verbal violence (60.8%), the threat of violence (42.1%), separation or divorce (38.8%), and physical abuse (23.1%).^3^,^7^,^11^,^12^ All these previous studies show outcomes that are similar to our findings.

Infertility introduces many factors that may induce or increase violence. Depending on cultural differences within communities, the proportion of women who suffer from violence and the factors affecting that violence may differ.^13^ In the present study, the women’s mean scale score was closer to the minimum score (38.74±11.48). Age, education, economic status, and the mother’s educational level have influenced the mean score of the scale. Kaur et al. (2014) reported that having insufficient economic means and illiteracy contribute to violence.^6^ In the Sheikhan et al. study (2014), a strong relationship was found between low income and domestic violence. Poverty and violence and a direct relationship between community and family were defined as the main factors underlying domestic violence toward women.^13^ However, it is reported that infertility affects the lives of millions of women worldwide regardless of their socioeconomic and educational levels.^3^ A significant difference was found between the mean score of scale and the treatment received previously, the present treatment, the duration of diagnosis and treatment, and IVF count. In Sheikhan et al. study (2014) found that the number of microinjections was associated with domestic violence.^13^ Some literature reported that the rate of domestic violence in those who had a prolonged duration of treatment was considerably higher.^1^,^14^ This demonstrates that long-lasting infertility and unsuccessful treatment cycles will intensify the stress that may lead to violence in marriage.^1^ But, interestingly, any difference between the mean scale score and from whom infertility was originated (p>0.05). Another study in our country found that 78% of infertile women had experienced domestic violence for the first time in the relationship with the current partner following diagnosis of female factor infertility.^8^ All countries and societies have norms embedded in the culture that may exacerbate gender-based violence.^15^ The environment where the victims live has a major role on potential outcomes of domestic violence.^16^ Violence is influenced by culture and ethnicity.^13^,^14^ Similarly, violence against women indicates that the male-dominated social structure has become more frequent.^14^ These results are thought to reflect regional differences between the eastern and western region of the country.

The mean score in the domestic violence domain was found to be close to the minimum value; infertile women most often agreed to the item of “it is insisted that I go to home visits with children, even though I do not want to take part.” Infertile individuals stated that they were extremely sensitive in discussions about children and pregnancy and that even in daily conversations they felt that people made comments that made fun of them.^10^ In a study by Berger et al. (2013), infertile women stated how it was difficult to see a new baby or a pregnant woman.^17^ In another study, it was reported that infertile women are generally excluded from daily events and celebrations related to mothers and children because it is believed that the children will be injured and affected by the evil eye of an infertile woman’s jealousy.^18^ In Turkish society, where women with children are cherished and those without children are evaluated with a negative/critical perspective, it could be asserted that infertile women avoid entering places full of children due to the feelings of shame and guilt caused by this situation.^19^

Women with infertility should be considered as an important group of often vulnerable patients with poor reproductive health who deserve attention and care in their own right.^20^ Even though it is frequently overlooked and not screened for, obstetricians and gynecologists who are advocates of women’s health should begin to screen for violence in infertile women.^21^ Therefore, counseling to avoid violence should be provided in infertility management, and health workers should be aware of gender-based violence. Hence, distinct psychosocial interventions should be developed and evaluated in infertility treatment, and psychological support for infertile women should be provided. Our results are important for reflecting domestic violence against infertile women and raising awareness of violence against infertile women in health personnel.

## CONCLUSION

From the findings of our study, it is pleasing to note that the infertile women in our study had a lower rate of being subjected to violence than those reported in other literature and studies in our country. However, although the violence rate was found to be lower, we also found that there was an increase in the incidence of violence against women due to infertility and that violence is experienced more once an infertility diagnosis has been made.

### Authors’ contribution

**RO, AT, SEG** made substantial contributions to the conception and design of the study, data acquisition and analysis, and drafted the manuscript.

**RO, SEG, BY** were involved in data analysis and in critically revising the manuscript.

All authors read and approved the final manuscript.
